# 674. Host and Pathogen Risk Factors for Fulminant *Clostridioides difficile* Infection

**DOI:** 10.1093/ofid/ofad500.736

**Published:** 2023-11-27

**Authors:** Hubert C Chua, Taryn A Eubank, Jinhee Jo, Kevin W Garey, Anne J Gonzales-Luna

**Affiliations:** Long Island University, Houston, Texas; University of Houston, Houston, Texas; University of Houston, Houston, Texas; University of Houston College of Pharmacy, Houston, TX; Department of Pharmacy Practice and Translational Research, University of Houston College of Pharmacy, Houston, Texas, USA, Houston, TX

## Abstract

**Background:**

Fulminant *Clostridioides difficile* infection (FCDI) is associated with high rates of mortality and morbidity; however, risk factors for FCDI are poorly understood. The objective of this study was to identify clinical and microbiological factors predictive of FCDI.

**Methods:**

This was a multicenter cohort study involving adult hospitalized patients diagnosed with an incident case of CDI (2015-22). Leftover stool samples were collected for ribotyping and determination of binary toxin presence (CDT; assumed presence in ribotypes 019, 027, 075, and 078-126). Disease severity was defined according to IDSA-SHEA CDI severity classification utilizing laboratory values obtained within 24 hrs of a positive CDI diagnostic test. FCDI was defined as CDI with hypotension (systolic blood pressure ≤ 90 mmHg), shock, ileus, or megacolon. Risk factors predictive of FCDI were assessed through multivariable logistic analysis.

**Results:**

Overall, 440 patients aged 62±18.2 years were identified (58.9% female). In univariate analysis, hypoalbuminemia (odds ratio [OR], 4.94; 95% confidence interval [CI], 2.94-8.31) and infection with a CDT-producing strain (OR, 1.99; 95% CI, 1.16-3.42) were associated with FCDI, and presence of both variables was associated with the highest odds of FCDI (OR, 5.73; 95% CI, 2.19-14.9). Using backwards stepwise logistic regression, CDI due to a CDT-producing strain and concomitant hypoalbuminemia was an independent predictor of FCDI (OR, 5.21; 95% CI, 1.97-13.8) after controlling for Charlson Comorbidity Index score >5 (OR, 1.85; 95% CI 0.99-3.48) and admission from a healthcare facility (OR, 1.73; 95% CI 1.02-2.95).

**Conclusion:**

Both clinical and microbiological factors were associated with increased odds of FCDI. Early identification of patients at high-risk for FCDI allows for more timely interventions to help mitigate the risk of disease progression. Future studies incorporating host microbiome features and novel biomarkers into CDI severity predictive models are needed.

**Disclosures:**

**Kevin W. Garey, MS;PharmD**, Paratek Pharmaceuticals: Grant/Research Support **Anne J. Gonzales-Luna, PharmD, BCIDP**, Cidara Therapeutics: Grant/Research Support|Ferring Pharmaceuticals: Personal Fees|Paratek Pharmaceuticals: Grant/Research Support|Seres Therapeutics: Grant/Research Support

